# Synthesis and Characterization of a Dual-Cation Organomontmorillonite Nanocomposite

**DOI:** 10.3390/ma11112320

**Published:** 2018-11-19

**Authors:** Guifang Wang, Huizhen Xiao, Shuai Zhang, Jun Qiu, Hengjun Li, Meijin Yang, Shaojian Ma, Sridhar Komarneni

**Affiliations:** 1 School of Resources, Environment and Materials, Guangxi University, Nanning 530004, China; wangguifang66@163.com (G.W.); xhz15777126431@163.com (H.X.); lhj18376721379@163.com (H.L.); meijinyang@sina.com (M.Y.); 2 Guangxi Key Laboratory of Processing for Non-Ferrous Metals and Featured Materials, Guangxi University, Nanning 530004, China; 3 Sinosteel Mining Company Limited, Sinosteel Corporation, Beijing 100080, China; zhangshuaiu521@163.com; 4 College of Chemical and Environmental Engineering, Shandong University of Science and Technology, Qingdao 266590, China; qiujun1968@163.com; 5 Materials Research Laboratory, Materials Research Institute, Pennsylvania State University, University Park, PA 16802, USA

**Keywords:** montmorillonite, modifier, adsorption, structural characteristics

## Abstract

In this study, a novel dual-cation organomontmorillonites (OMt) nanocomposite was synthesized by two kinds of modifiers cetyltrimethylammonium chloride and cysteamine hydrochloride, and the adsorption behavior of modifiers into montmorillonite (Mt) has been investigated. The OMt were characterized by techniques, such as X-ray diffraction (XRD), Fourier Transform Infrared (FTIR) spectroscopy, and thermogravimetric and differential thermal (TG-DTA) analyses. The effects of temperature, contact time, the order of addition and the concentration of organic modifiers on the amounts of organics adsorbed were investigated. The adsorption amount of cetyltrimethylammonium chloride (CTAC) and cysteamine hydrochloride (CSH) increased with the increase of the added CTAC amount and contact time, while the addition order of modifiers and modification temperature had no significant effect on the actual adsorption amount of CTAC and CSH on Mt, as confirmed by the XRD patterns. The experimentally determined isotherms showed a good fit with the Langmuir adsorption models. The adsorption kinetics demonstrated that the adsorption of CTAC and CSH by Mt followed the pseudo-second-order model, and CTAC adsorption rate on Mt was faster than that of CSH. FTIR spectrum clearly revealed the incorporation of surfactant ions into the interlayer region. The TG-DTA analyses showed that the total mass losses of OMt strongly depended on the molecular volume of modifiers.

## 1. Introduction

Montmorillonite (Mt) is a 2:1 type of layered silicate mineral composed of two silica tetrahedral sheets (SiO_4_) sandwiching an aluminum octahedral sheet [Al_2−x_ Mg_x_O_4_(OH)_4_]. Mt is negatively charged due to the isomorphous replacement of mainly Mg^2+^ for Al^3+^ in the octahedral sheet [1,2,3,4]. Therefore, the natural Mt can attract species with opposite charges, such as heavy metal ions and organic cations, and it also shows high specific surface area, high cation exchange capacity (CEC), and micro and meso-porosity properties [5,6]. Based on these unique characteristics as well as their natural abundance and cost-effectiveness, Mt is usually used as a low cost adsorbent in wastewater treatment. Because of its negative charge, Mt is capable of efficiently adsorbing heavy metal ions by the cation exchange process in the interlayer space of Mt, but it shows low affinities to non-ionic and non-polar organic compounds (NOCs) [7,8]. However, the affinity and the adsorption capacity for NOCs can be improved by introducing organic cations, such as quaternary ammonium cations into the interlayers of Mt, i.e., by producing organomontmorillonite (OMt). Most previous research has shown that the OMt has high affinity toward hydrophobic organic compounds and it can be more effective in the adsorption of organic pollutants, such as phenol, *p*-chlorophenol, benzene, and toluene [9,10,11].

However, in the real world, such as the wastewater discharge in the mineral processing of nonferrous metals, the organic pollutants usually exist with heavy metal ions and therefore, effective and low-cost adsorbents for removing both heavy metals and organic contaminants are highly needed. Previous studies showed that the type and structure of organic modifiers such as various cationic surfactants with different chain lengths and branched-structure can remarkably affect the textural properties, thermal stability, and adsorptive behavior of OMt [12,13,14,15]. Moraru [16], Khatib et al. [17], and Özkan Açışlı et al. [18], for example, used different quaternary ammonium salts to prepare single-cation OMt and found that the adsorption capacity of the surfactants adsorbed onto Mt, the interlayer distance and the packing density of surfactant ions in the interlayer increased with the increase of alkyl chain lengths. Yang et al. [19] studied the adsorption of cetyltrimethylammonium cations by montmorillonite, indicating that the adsorption reaction was carried out rapidly at room temperature, and the adsorption process was in accordance with the Freundlich isothermal type. Therefore, if the organic modifier for intercalating into the interlayer of Mt contains both quaternary ammonium cations and the chelating groups, such as –SH, –COOH, and –NH_2_, the resulting OMt might serve as an effective adsorbent for simultaneously removing organic contaminants as well as heavy metals from wastewater [20]. Dual-cation OMt, a relatively novel type of modified Mt prepared by the intercalation of two types of cationic surfactants, such as trimethyltetradecyl ammonium chloride or hexadecyltrimethyl ammonium chloride or octadecyltrimethyl ammonium chloride and cysteamine hydrochloride (CSH) have been previously reported to effectively remove both heavy metal ions and organic pollutants because the functional groups, including quaternary ammonium cations and the –SH group of CSH have the ability to interact with organic compounds and heavy metal ions by the electrostatic interaction, formation of complexes, hydrophobic interactions and/or partitioning actions in an organic phase [20]. However, with respect to the dual-cation OMt, the adsorption behavior of two different types of organic modifiers by Mt from aqueous solutions was not clear from the previous studies, and further investigations are needed to explore the influence of the types of modifiers and intercalation methods on the adsorption ability of modifiers by Mt and the structural characteristics of dual-cation OMt.

Therefore, in this work, the dual-cation OMt was synthesized with cetyltrimethylammonium chloride (CTAC) and cysteamine hydrochloride (CSH) using three different intercalation methods with different molar ratios of CTAC/CSH: (1) CSH was intercalated into Mt after CTAC, (2) CTAC was intercalated into Mt after CSH, and (3) CTAC and CSH were used simultaneously for the intercalation. The influence of intercalation methods and molar ratio of modifiers on the amount of modifiers adsorbed by Mt was investigated. The interlayer spacing, aggregation form of the adsorbed organic cations and the thermal stability of the dual-cation OMt were analyzed with X-ray diffraction (XRD), Fourier transform infrared spectra (FTIR), and thermogravimetric and differential thermal (TG-DTA) analysis experiments. The adsorption mechanism of modifiers on Mt was also discussed from the aspects of adsorption kinetics and thermodynamics.

## 2. Experimental Part

### 2.1. Materials

The raw montmorillonite (Mt) that was used in this study was obtained from Tiandong bentonite deposit in Guangxi province of China. The sample was purified by natural sedimentation and the <10 μm fraction was collected and dried at 95 °C. The collected montmorillonite contains very low concentrations of feldspar and quartz. The sample was ground and sieved through 200 mesh and then Na^+^ exchanged by treating with Na_2_CO_3_. The obtained Na^+^ exchanged Mt (denoted as Na^+^-Mt) sample was used in all subsequent experiments for the preparation of organic modified montmorillonite (OMt). The cation exchange capacity (CEC), methylene blue adsorption, and the swelling volume of Na^+^-Mt were measured to be 120.0 mmol/100 g, 37.0 g/100 g, and 56.0 mL/g, respectively. The following reagents were used for modification of the montmorillonite: cetyl (hexadecyl) trimethylammonium chloride (CTAC, 97%, analytical grade), and complexant, cysteamine hydrochloride (CSH, 98%, analytical grade).

### 2.2. Adsorption of Organic Modifiers into Mt and Preparation of OMt

Batch experiments, including the influence of heating temperature, contact time, added amount, and order of addition of modifiers were conducted to determine the adsorption characteristics of montmorillonite towards organic modifiers. In a typical run, different amounts of CTAC and CSH were added into the Na^+^-Mt dispersion in iodine flask under fast stirring and then the mixture was heated at 40–60 °C for 2–120 min to facilitate the intercalation of organic modifiers into the interlayer spaces of Na^+^-Mt. The treated dispersions were centrifuged and washed with distilled water several times, and then the products were dried, ground, and sieved to obtain <74 μm size prior to characterization and analyses of organic carbon (f_oc)_ and sulfur (f_os_) contents.

Three different intercalation methods were explored to synthesize OMt and investigate the effects of added amount and order of addition of modifiers, as follows: (1) Different amounts of CTAC were first added into the Na^+^-Mt dispersion with continued stirring for 2 h, and then the controlled amount of CSH equal to the CEC of Na^+^-Mt was added into the dispersion for intercalation; (2) Different amounts of CSH were first added into the Na^+^-Mt dispersion, and then the controlled amount of CTAC equal to the CEC of Na^+^-Mt was added into the solution for intercalation; (3) CTAC and CSH with different amounts were simultaneously added into the Na^+^-Mt dispersion to synthesize OMt by intercalation. When the amounts of CTAC was fixed at 1.0 CEC of Na^+^-Mt, the amounts of the other modifiers CSH were selected as 0, 0.25, 0.5, 1.0, 1.5, and 2.0 CEC of Na^+^-Mt to obtain different molar ratios of modifiers and vice versa. For each case, the stirring speed was 180 r/min, and the modification temperature was 40 °C. The OMt obtained from the former two methods were denoted as CTAC_x_-CSH-Mt and CSH_x_-CTAC-Mt, respectively, and those OMt obtained using the third method were designated as CTAC_x_-CSH-coMt or CTAC-CSH_x_-coMt (here “x” indicates the added amounts of CTAC or CSH for synthesizing OMt, which were 0.25, 0.5, 1.0, or 2.0). For the purpose of comparison, the CSH modified montmorillonite (CSH-Mt) and CTAC intercalated montmorillonite (CTAC-Mt) were also synthesized. The heating temperature was controlled at 40 °C, 50 °C, and 60 °C using only the third intercalation method and at predetermined time intervals of 2, 4, 6, 10, 30, and 120 min to investigate the adsorption isotherms and kinetics, respectively. The adsorption of organic modifiers onto Mt was characterized by the CTAC or CSH loading amounts, which was calculated from the obtained f_oc_ and f_os_ values.

### 2.3. Desorption of Organic Modifiers from OMt

The desorption experiments of OMt were conducted, as follows: 0.5 g of CTAC-CSH-coMt powder sample was dispersed in 250 mL conical flasks to which 50 mL distilled water solution with the initial pH ranges of 3.0–11.0 were added. The dispersions were shaken at 25 °C under fast stirring for 2 h, and then centrifuged to separate solution from solid. The obtained solid products were dried, ground, and sieved to obtain <74 μm size prior to the analyses of organic carbon (f_oc_) and sulfur (f_os_) contents. The desorption rates (η) of organic modifiers from the CTAC-CSH-coMt sample were calculated using the following equation:

(1) $$\;\eta (\%)\;=\;[(Q_{t}\;-\;Q_{0})/Q_{t}]\;\times \;100\%\;$$

where *Q_t_* and *Q*_0_ (mmol·g^−1^) are the loading amounts of CTAC or CSH before and after the desorption of OMt.

### 2.4. Characterization

Powder X-ray diffraction (XRD) data were recorded with a RigaKu D/max-rB diffractometer (Rigaku Corporation, Akishima-shi, Japan) using Cu Kα radiation at 40 kV and 100 mA. The functional groups present in the montmorillonite samples were identified by using the NICOLET 5700 Fourier infrared spectrometer (Thermo Nicolet Corporation, Madison, SD, USA) with the KBr pellet method. Thermogravimetric and differential thermal analysis (TG-DTA) were performed on a TAQ600 thermal analyzer (TA Instruments, Newcastle, PA, USA) at the heating rate of 20 °C/min from 30 to 1000 °C. Organic carbon (f_oc_) and sulfur (f_os_) contents of the original and modified Mt were examined with a Vario EL III elemental analyzer (Elementar Analysensysteme, Hanau, Germany).

## 3. Results and Discussion

### 3.1. Effect of the Order of Addition and Amount of Added Modifiers on Their Adsorption by Mt

**Figure 1:**
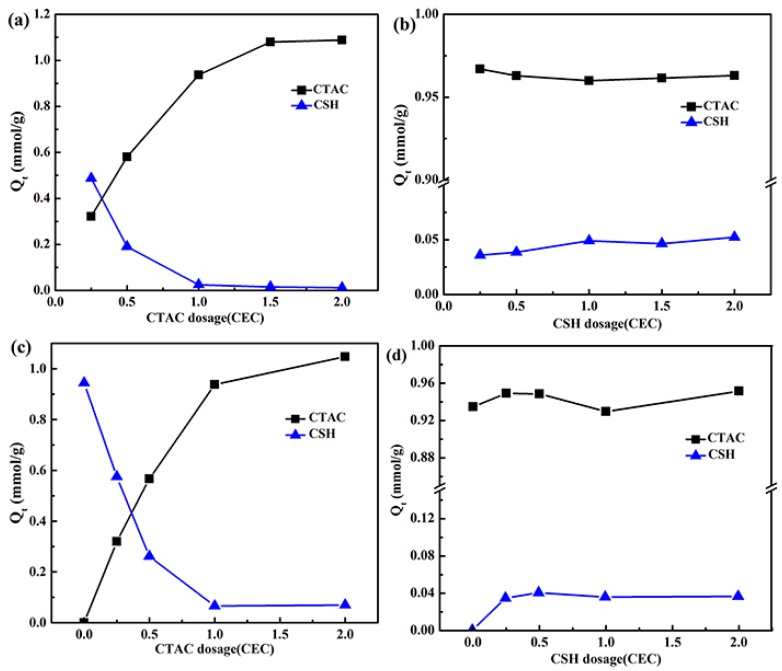
Effect of the order of addition and the mount of added modifiers on the CTAC or CSH adsorption amounts (*Q_t_*) by different samples: (**a**) CTAC_x_-CSH-Mt, (**b**) CSH_x_-CTAC-Mt, (**c**) CTAC_x_-CSH-coMt, and (**d**) CTAC-CSH_x_-coMt.

For all the above prepared CTAC_x_-CSH-Mt, CSH_x_-CTAC-Mt, CTAC_x_-CSH-coMt, and CTAC-CSH_x_-coMt samples, the CTAC or CSH adsorption amounts (*Q_t_*) by Mt are shown in Figure 1. The *Q_t_* was calculated from the organic carbon (f_oc_) and sulfur (f_os_) contents, which were determined by the elemental analyzer (Elementar Analysensysteme, Hanau, Germany).

As can be seen from Figure 1, for the OMt samples that were prepared by three different methods, when the addition amount of CSH was fixed to 1.0 CEC of Na^+^-Mt, the adsorption amount of CTAC on Mt remarkably increased as the CTAC dosage increased, while the CSH adsorption amount decreased. Also, as for the CTAC_x_-CSH-Mt samples, when the addition amount of CTAC increased to 1.5 CEC of Na^+^-Mt, the maximum adsorption amount of CTAC occurred and there was almost no further increase in adsorption beyond this addition amount (Figure 1a). However, when the addition amount of CTAC was fixed to 1.0 CEC of Na^+^-Mt, the CSH and CTAC adsorption amounts on Mt did not change significantly, but the CTAC adsorption amount was much larger than that of CSH. This suggested that the effects of the CTAC and CSH amounts on the adsorption amount of the modifiers was dominated by the added amount of CTAC. Moreover, when the amount of the two modifiers was 1.0 CEC of Na^+^-Mt, for CTAC-CSH-coMt, CTAC_1.0_-CSH-Mt, and CSH_1.0_-CTAC-Mt samples, the CTAC adsorption amounts on Mt were about 0.936, 0.936, and 0.960 mmol/g, respectively, while those of CSH were 0.06, 0.02, and 0.048 mmol/g, respectively (Figure S1), which indicated that the different addition order of modifiers had no significant effect on the actual adsorption amount of CTAC and CSH on Mt. It appears that the CTAC with longer alkyl ammonium chains could easily enter into the interlayers than CSH. The pre-intercalated CTAC could easily “block” the interlayer spaces with its relatively large adsorption amounts, and therefore, CSH could not be further intercalated easily into the interlayers [21]. Also, the pre-intercalated CSH in the interlayer spaces could be replaced by post-intercalated CTAC because of its larger volume/charge ratio than that of CSH, and thus the amount of CSH entering into the interlayers of montmorillonite was relatively small. When considering that, among all of the adsorbents CTAC-CSH-coMt, CTAC_1.0_-CSH-Mt, and CSH_1.0_-CTAC-Mt, the actual adsorption amounts of CTAC or CSH on Mt were similar, CTAC-CSH-coMt was chosen to investigate in detail with further adsorption experiments.

### 3.2. Effect of Modification Temperature and Time on Adsorption of Organic Modifiers by Mt

**Figure 2:**
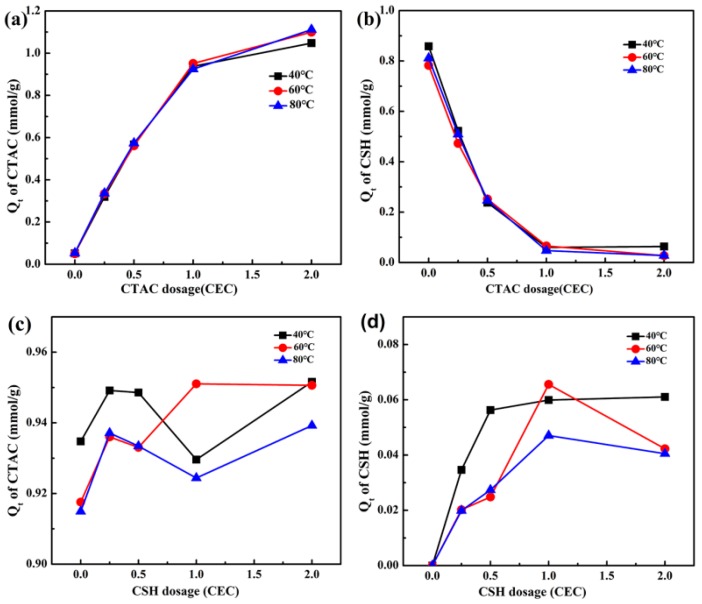
Effect of modification temperature on the adsorption amount (*Q_t_*) of cetyltrimethylammonium chloride (CTAC) (**a**,**b**) and cysteamine hydrochloride (CSH) (**c**,**d**) under the condition of different dosages of modifiers.

**Figure 3:**
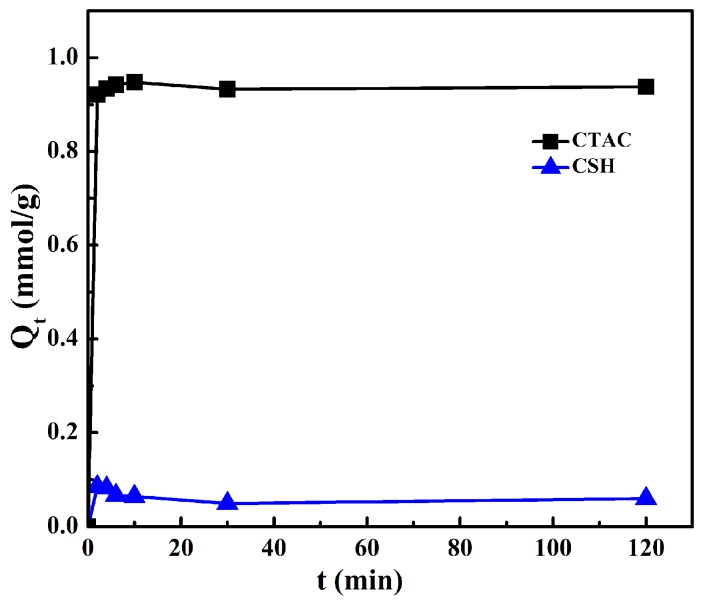
Effect of time on the adsorption amount (*Q_t_*) of modifiers.

For sample CTAC-CSH-coMt, the effects of the modification temperature and contact time on the actual adsorption amounts of CTAC and CSH by Mt are presented in Figure 2 and Figure 3, respectively.

It can be seen from Figure 2 that the modification temperature had little effect on the uptake of CTAC and CSH on Mt under the conditions of different CTAC or CSH dosages. However, Figure 3 showed that the modification time could evidently influence the adsorption amount of CTAC and CSH on Mt, and the process of themodifier entering into the montmorillonite interlayer can be divided into three stages. In the first stage, the adsorption amount of the modifiers on Mt increased rapidly and the adsorption rate was very fast. At 2 min, the adsorption amount of CTAC onto Mt reached 0.92 mmol/g, which was equal to 0.77 CEC of Na^+^-Mt. The adsorption amount of CSH also reached the maximum of 0.084 mmol/g, but the adsorption efficiency was relatively low in comparison with that of CTAC. This could be attributed to the competitive adsorption between the two modifiers of CTAC and CSH with different molecular volumes. At the second stage of 2–10 min, the adsorption amount of CTAC showed a slow growth trend with the increase of equilibration time, while the uptake of CSH changed only a little. After 10 min, the adsorption amounts of the organic modifiers of CTAC and CSH on Mt had no significant change and they apparently reached equilibrium.

### 3.3. Adsorption Kinetics

The adsorption kinetics were mainly used to evaluate the adsorption efficiency between the adsorbent and the adsorbate [22], and it describes the relationship between adsorption time and adsorption rate. The pseudo-second-order kinetic equation that was used in this study was derived by Ho [23] from adsorption of divalent metal ions. The equation was given, as follows [23]:

(2) $$\;\frac{dq_{t}}{dt}=k_{2}{\left(q_{e}-q_{t}\right)}^{2}\;\;$$

(3) $$\;\frac{t}{q_{t}}=\frac{1}{k_{2}q_{e}{}^{2}}+\frac{t}{q_{e}}\;\;$$

where *q_t_*, *q_e_* (mmol·g^−1^) was the adsorption amount at time *t* and equilibrium time, respectively. *k*_2_ (g·mol^−1^·min^−1^) was pseudo-second-order kinetics rate constant.

**Figure 4:**
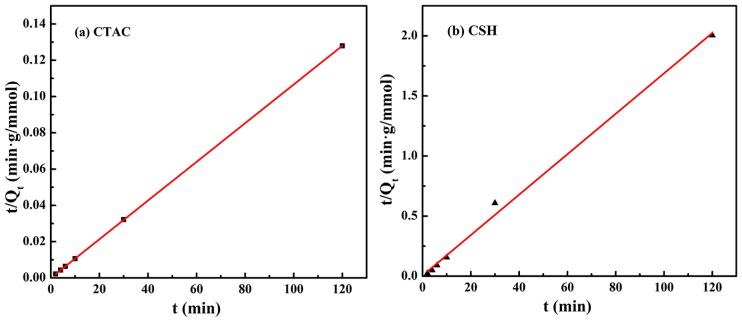
Pseudo-second-order model plots of CTAC (**a**) and CSH (**b**) onto Mt.

In order to determine the adsorption mechanism of modifier CTAC and CSH on Mt, the pseudo-second-order kinetics model was used in this study to simulate the adsorption data of CTAC and CSH on Na^+^-Mt at different contact times (Figure 3). The parameters *k*_2_ and *q_e_* can be obtained by the intercept and slope of *t*/*q_t_* versus t. The fitting results that were obtained from this kinetics equation are shown in Figure 4, and the corresponding kinetic parameters *q_e_*, *k*_2_ and the correlation coefficient *R*^2^ are summarized in Table 1.

As can be seen from Figure 4 and Table 1, the correlation coefficients, *R*^2^ values calculated from the fitting of the kinetics equation were > 0.99, and the experimental value of equilibrium adsorption *q_e_* (*exp*) showed good agreement with the calculated ones *q_e_* (cal), which indicated that the adsorption and intercalation process of CTAC and CSH onto Mt can be well fitted with the pseudo-second-order model. Additionally, the adsorption rate constant *k*_2_ of CTAC and CSH on Mt were 0.13285 and 0.04748 g·mol^−1^·min*^−^*^1^, respectively. The former was found to be three times as large as the latter, indicating that the CTAC adsorption rate on Mt was faster than that of CSH, which may be related to the carbon chain length and hydrophobicity of the organic modifiers.

### 3.4. Adsorption Isotherms

In order to gain better understanding of the interaction mechanism between modifiers and Mt, both Langmuir and Freundlich adsorption isotherm models in their non-leaner forms were applied to the loading amounts of CTAC and CSH on Mt. The Langmuir and Freundlich adsorption models are expressed, as follows [24,25,26]:

(4) $$\;q_{e}=\frac{QbC_{e}}{1+bC_{e}}\;\;$$

(5) $$\;q_{e}=K_{F}c_{e}{}^{1/n}\;\;$$

where *q_e_* (mmol·g^−1^) represents the adsorption capacity of unit molar adsorbent, *Q* (mmol·g^−1^) is the maximum adsorption capacity of adsorbent to form a complete monolayer coverage on the surface, and *C_e_* (mmol·L^−1^) is the concentration of the adsorbate ion solution at equilibrium. *b* (L·mmol^−1^) is the Langmuir constant related to the energy of adsorption, *K_F_* and *n* are the Freundlich constant related to the adsorption capacity and adsorption strength of the adsorbent, respectively.

**Figure 5:**
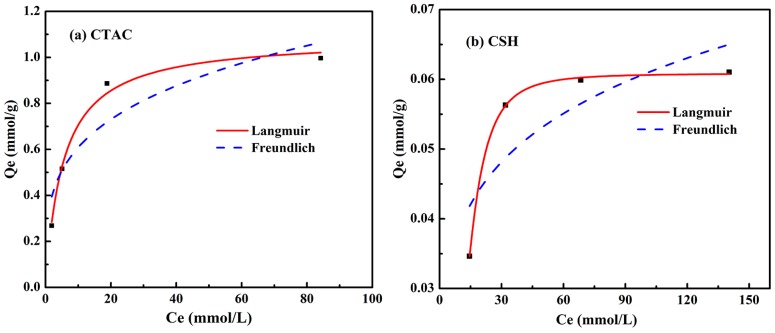
Adsorption isotherms of CTAC (**a**) and CSH (**b**) at 40 °C for Mt.

The experimental data in Figure 2a,d were fitted using the Formulas (4) and (5) given above, and the corresponding isotherm plots and parameters are shown in Figure 5 and Table 2, respectively.

It can be seen from Figure 5 and Table 2 that the adsorption isotherms of CTAC and CSH by Mt can be well fitted with Langmuir model, which is in agreement with the high correlation coefficients (*R*^2^  >  0.99). This indicated that the available active sites on the surface of organic modified Mt were homogenously distributed and the adsorption process of CTAC onto OMt was of the monolayer type. According to Table 2, the calculated maximum adsorption capacity (*Q*_m_) of CTAC and CSH by Mt were 1.0862 and 0.06082 mmol·g^−1^, respectively, which was slightly larger than the actual adsorption amount of CTAC and CSH onto Mt when the molar ratio of CTAC/CSH was 2:1.

### 3.5. Desorption Studies of Modified Mt

To investigate the stability of OMt and the interaction extent between the organic modifiers and Mt, the desorption experiments of CTAC and CSH from OMt were carried out and the effects of the initial pH of the water solution on the desorption rates of CTAC and CSH from the CTAC-CSH-coMt sample are presented in Table 3.

As shown in Table 3, when the initial pH values of the water solution were 3.0, 7.0, and 11.0, the desorption rates of CTAC and CSH from the CTAC-CSH-coMt sample were at the range of 0.72–1.29%, indicating that the composite modified Mt was relatively stable under different pH value, and there were the strong interaction between the organic modifiers and Mt. Additionally, in the pH range of 3.0–11.0, and the minimum desorption rates of CTAC and CSH were found at pH 11.0, which suggested that the alkaline environment was helpful for the stability of OMt.

### 3.6. Characterization Results

**Figure 6:**
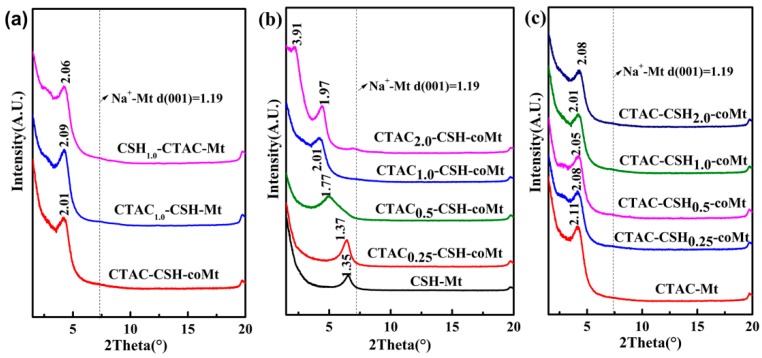
X-ray diffraction (XRD) patterns of OMt obtained under three different preparation methods: different order of addition using the same amount of CTAC and CSH (**a**), with different amounts of CTAC (**b**) and CSH (**c**).

Powder X-ray diffraction results of composites of modified montmorillonite products under different CTAC or CSH dosage conditions and three different preparation methods are shown in Figure 6.

As can be seen from Figure 6, the layer spacing *d*_(001)_ values of all the Mt samples that were modified by CTAC and/or CSH were larger than that of unmodified Na^+^-Mt, which indicated that the basal spacing was enlarged by the intercalation of organic cations into the interlayer region of Mt. Also, the basal spacing generally depended on the loading amount and orientation in the interlayer galleries of the organic cations [27,28]. Figure 6a shows that the *d*_(001)_ diffraction peaks of CTAC_1.0_-CSH-coMt, CTAC_1.0_-CSH-Mt and CSH_1.0_-CTAC-Mt samples obtained under three different preparation methods showed similar *d*_(001)_ values of 2.01 nm, 2.09 nm, and 2.06 nm, respectively. This means that the addition methods of two modifiers had little effect on the intercalation of CTAC and CSH into Mt, which was in good agreement with the results of the CTAC and CSH adsorption amounts for the above three samples (Figure S1).

Figure 6b presents the XRD results of CSH-Mt and CTAC-CSH-coMt samples that were prepared under different used amounts of CTAC. It showed that the peak in the patterns of CTAC_0.25_-CSH-coMt corresponded to 1.37 nm interlayer spacing value, which was a little larger than that of the CSH-Mt sample. While with the increase of the added amounts of CTAC, the peaks with larger 2θ value gradually shifted to smaller 2θ value, and the corresponding layer spacing *d*_(001)_ values increased from 1.37 nm to 2.01 nm. As the used amounts of CTAC increased to 2.0 CEC of Na^+^-Mt, the CTAC_2.0_-CSH-coMt samples showed double peaks in the patterns, corresponding to the layer spacing *d*_(001)_ values of 1.97 nm and 3.91 nm, respectively, which indicated that at the added CTAC amounts of 2.0 CEC of Na^+^-Mt, the interlayer of Mt could be expanded to a large extent and the layer spacing *d*_(001)_ values highly depended on the used amounts of CTAC.

The XRD patterns of CTAC-Mt and CTAC-CSH-coMt samples that were synthesized under different added amounts of CSH are presented in Figure 6c. It showed that all of the samples had similar layer spacing and the added CSH did not expand the interlayers of CTAC-Mt further, which was consistent with the results of similar CSH adsorption amounts for CTAC-CSH-coMt samples that were synthesized under different added amounts of CSH (Figure 1d).

**Figure 7:**
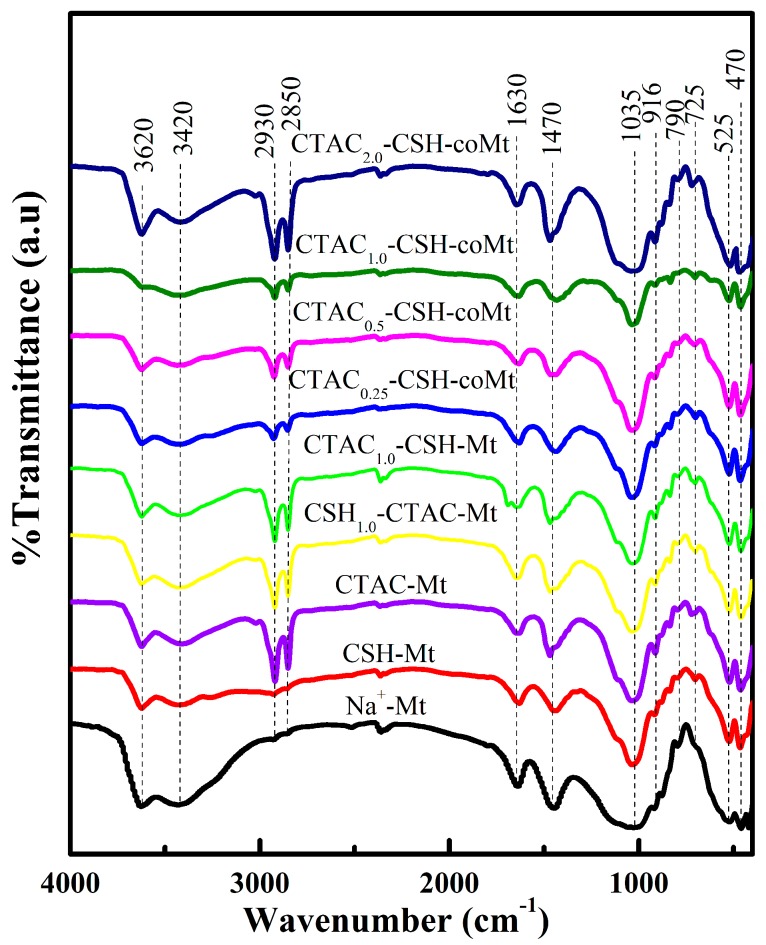
Fourier transform infrared spectra (FTIR) spectra of Na^+^-Mt and different OMt.

FTIR is a useful way to investigate the aggregation form of the adsorbed organic cations on Mt. The FTIR spectra of compoud OMt samples prepared using different amounts of organics and using different addition order of CTAC and CSH are shown in Figure 7.

The CTAC-Mt, CSH-Mt and all CTAC_1.0_-CSH-Mt complexes displayed characteristic vibrations at about 2930 cm^−1^ for asymmetric CH_2_ stretching mode and 2850 cm^−1^ for symmetrical CH_2_ stretching mode (Figure 7) [29,30,31]. It can be seen that the intensities of the vibrations strongly depended on the amounts of CTAC used and the order of additions of the two modifiers. With the increase of the added CTAC amounts, the asymmetric and symmetrical CH_2_ stretching mode became well resolved and their intensities clearly increased. The bands around 1630 cm^−1^ are assigned to the ν_2_ (H–O–H) bending vibration and the frequency shifts from 1623.29 cm^−1^ (CTAC_0.25_-CSH-coMt) to 1639.98 cm^−1^ (CTAC_2.0_-CSH-coMt) (Table S1), which is similar to that of organomontmorillonite that was reported in previous studies [18,31,32]. This indicates that the hydrophobicity of the CTAC-CSH-coMt complexes increases with the increased amount of surfactant adsorption.

**Figure 8:**
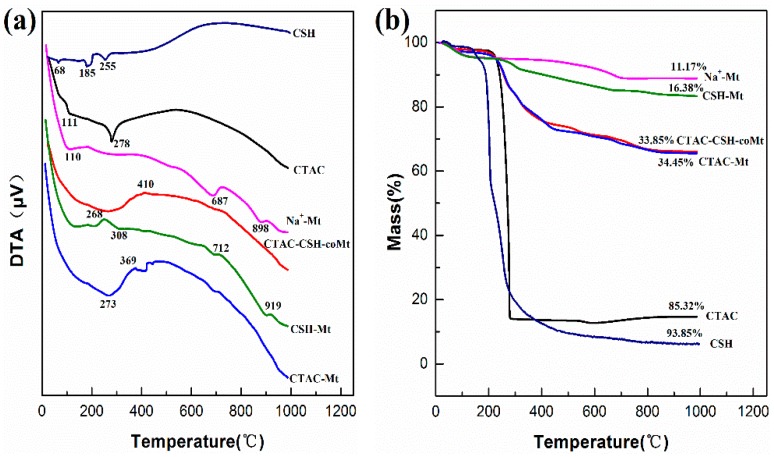
Differential thermal analyses (DTA) (**a**) and thermogravimetric (TG) (**b**) curves of CTAC, CSH, Na^+^-Mt, and OMt.

The thermogravimetric and differential thermal (TG-DTA) analyses of CTAC, CSH, Na^+^-Mt, and OMt are shown in Figure 8.

It can be seen from Figure 8 that the mass loss at the 30~135 °C temperature range corresponded to the evaporation of adsorbed water from the Mt surfaces and water molecules around the exchangeable cations [33]. As for the pure organic modifiers CSH and CTAC, the main mass loss occurred at 185, 255, and 278 °C, respectively (Figure 8). For CTAC-Mt, CSH-Mt, and CTAC_1.0_-CSH-Mt samples, the mass loss occurred between 150 and 500 °C and this was mainly due to the thermal decomposition of CTAC and CSH. The mass losses for all the samples in the temperature range of 500~900 °C, were attributed to the dehydroxylation of OH groups in the aluminosilicate structure of Mt [34,35]. Moreover, it can be found that the total mass losses of all the above OMt samples were larger than that of Na^+^-Mt by itself, and the total mass losses of OMt increased in the following order: CTAC-Mt (34.45%) > CTAC-CSH-coMt (33.85%) > CSH-Mt (16.38%), which indicated that the total mass losses of OMt strongly depended on the molecular volumes of organic modifiers.

## 4. Conclusions

Cetyltrimethylammonium chloride and cysteamine hydrochloride were used to modify montmorillonite (Mt), and the effects of temperature, contact time, the addition order of modifiers and modification temperature were investigated by using a batch process-type of experiments. The results showed that the adsorption amounts of the organic modifiers and layer spacing d_(001)_ values were highly dependent on the amounts of CTAC used. The addition order of modifiers and modification temperature had no significant effect on the actual adsorption amount of CTAC and CSH on Mt. The pre-intercalated CTAC could ‘‘block” the interlayer spaces with its relatively large adsorption amounts, and therefore, CSH could not be further intercalated easily into the interlayers. The adsorption equilibrium was achieved in 10 min, as determined by kinetics experiments. The adsorption kinetics demonstrated that the adsorption and intercalation process of CTAC and CSH onto Mt can be well fitted with the pseudo-second-order model, and CTAC adsorption rate on Mt was faster than that of CSH. The adsorption isotherms were determined and simulated using Langmuir and Freundlich models, and Langmuir model could represent the experimental data well, which indicated that the available active sites onto the surfaces of modified Mt with organic were homogenously distributed and the adsorption process of CTAC and CSH onto OMt was of the monolayer-type. The thermogravimetric and differential thermal (TG-DTA) analysis of Na^+^-Mt and OMt showed that the total mass losses of OMt were larger than that of Na^+^-Mt, and the total mass losses of OMt strongly depended on the molecular volumes of organic modifiers.

## Figures and Tables

**Table 1:** Kinetic parameters of CTAC and CSH adsorption onto Mt.

Organic Modifiers	*q_e,exp_*/mmol·g^−1^	*q_e,cal_*/mmol·g^−1^	*k*_2_/g·mol^−1^·min^−1^	*R* ^2^
CSH	0.05989	0.05945	0.04748	0.995
CTAC	0.93772	0.93758	0.13285	0.999

**Table 2:** Langmuir and Freundlich isotherm parameters for adsorption of CTAC and CSH onto Mt.

Modifiers	Langmuir Model			Freundlich Model		
	*Q_m_*/mmol·g^−1^	*b*/L·mmol^−1^	*R* ^2^	*K* *_F_*	*n*	*R* ^2^
CTAC	1.0862	0.1855	0.99	0.33281	3.8104	0.86
CSH	0.06082	0.00069	0.99	0.02484	5.14034	0.56

**Table 3:** The desorption results of organic modifiers from organomontmorillonite (OMt) under different initial pH value.

Initial pH Value	Desorption Rate of CSH/%	Desorption Rate of CTAC/%
3.0	0.87	1.15
7.0	1.29	0.91
11.0	0.85	0.72

## Supplementary Materials

The following are available online at http://www.mdpi.com/1996-1944/11/11/2320/s1, Figure S1: Effect of the order of addition and the amount of modifiers on the CTAC or CSH amount adsorbed onto the OMt samples prepared by the three different intercalation methods when the amounts of STAC and CSH was fixed at 1.0 CEC, Table S1: Positions and assignments of the FTIR vibration bands in the range of 400–4000 cm^−1^.
