# Supplementary material for: Synthesis and Characterization of a Dual-Cation Organomontmorillonite Nanocomposite
Source: Materials (Basel). 2018 Nov 19;11(11):2320. doi: 10.3390/ma11112320 (PMC6265940; doi:10.3390/ma11112320)
Supplement: Supplementary file 1 [file materials-11-02320-s001.pdf]

Synthesis and characterization of a dual-cation organomontmorillonite nanocomposite

Guifang Wang [a,b], Huizhen Xiao [a], Shuai Zhang [c], Jun Qiu [d], Hengjun Li [a], Meijin Yang [a], Shaojian Ma [a,*], Sridhar Komarneni [e,*]

[a]School of Resource Environment and Materials, Guangxi University, Nanning 530004, China.

[b]Guangxi Key Laboratory of Processing for Non-ferrous Metals and Featured Materials, Guangxi University, Nanning 530004, China.

[c]Sinosteel Mining Company Limited, Sinosteel Corporation, Beijing 100080, China.

[d]College of Chemical and Environmental Engineering, Shandong University of Science and Technology, Qingdao 266590, China.

[e]Materials Research Laboratory, Materials Research Institute, Pennsylvania State University, University Park, PA 16802, USA

Supporting Information:

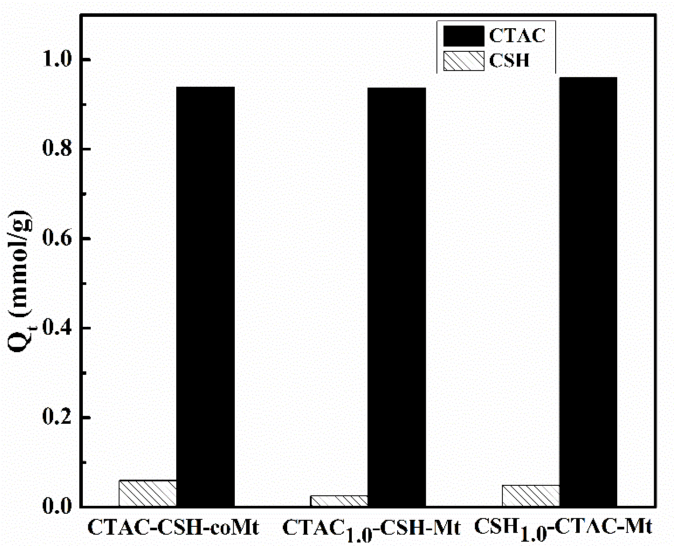

Fig. S1 Effect of the order of addition and the amount of modifiers on the CTAC or CSH amount adsorbed (Q_t) onto the OMt samples prepared by the three different intercalation methods when the amounts of STAC and CSH was fixed at 1.0 CEC.

Table S1. Positions and assignments of the FTIR vibration bands in the range of 400-4000 cm^{-1}

Assignments	Structural OH stretching	Symmetric OH stretching	N-H stretching	Asymmetric CH_2 stretching	Symmetrical CH_2 stretching	H–O–H bending	CH_2 shearing	Si-O stretching	CH_2 rocking	C-S stretching
Na^+-Mt	3630.82	3452.49	--	--	--	1636.85	1448.09	1036.15	794.21	--
CSH-Mt	3621.44	3447.28	3249.96	2921.17	2860.14	1625.38	1441.83	1029.90	796.30	703.48
CTAC-Mt	3624.57	3424.33	--	2921.66	2851.80	1639.98	1469.99	1033.02	793.16	--
$CSH_{1.0}$-CTAC-Mt	3621.44	3444.15	--	2919.58	2848.67	1645.19	1467.90	1038.24	788.99	697.22
$CTAC_{1.0}$-CSH-Mt	3624.57	3427.46	--	2921.67	2851.80	1631.63	1469.99	1036.16	792.12	700.35
$CTAC_{0.25}$-CSH-coMt	3624.57	3435.81	3243.70	2924.80	2854.92	1623.29	1441.83	1036.16	796.23	694.01
$CTAC_{0.5}$-CSH-coMt	3627.70	3432.68	3247.87	2924.78	2851.80	1633.72	1464.77	1036.16	794.21	703.48
$CTAC_{1.0}$-CSH-coMt	3616.22	3429.56	--	2921.67	2854.92	1636.85	1431.40	1033.02	792.12	705.56
$CTAC_{2.0}$-CSH-coMt	3619.35	3421.21	--	2919.58	2851.80	1639.98	1470.00	1024.68	790.00	717.03
